# Acute management of stroke in Iran: Obstacles and solutions

**Published:** 2017-04-04

**Authors:** Shima Shahjouei, Reza Bavarsad-Shahripour, Farhad Assarzadegan, Reza Rikhtegar, Masoud Mehrpour, Babak Zamani, Georgios Tsivgoulis, Andrei Alexandrov, Anne Alexandrov, Ramin Zand

**Affiliations:** 1 Department of Neurosurgery, School of Medicine, Tehran University of Medical Sciences, Tehran, Iran; 2 Department of Neurology, University of Alabama, Birmingham, Alabama, USA; 3 Department of Neurology, School of Medicine, Shahid Beheshti University of Medical Sciences, Tehran, Iran; 4 Neuroscience Research Center, Tabriz University of Medical Sciences, Tabriz, Iran; 5 Department of Neurology, School of Medicine, Iran University of Medical Sciences, Tehran, Iran; 6 Department of Neurology, University of Tennessee Health Science Center, Memphis, Tennessee, USA; 7 Second Department of Neurology, Attikon University Hospital, School of Medicine, University of Athens, Athens, Greece; 8 Department of Neurology, University of Tennessee Health Science Center, Memphis, Tennessee, USA; 9 Department of Neurology, Geisinger Health System, Danville, Pennsylvania, USA

**Keywords:** Stroke, Thrombolytic Therapy, Tissue Plasminogen Activator, Hospital Rapid Response Team, Quality Improvement, Iran

## Abstract

**Background:** Stroke is among the leading causes of mortality and permanent disability in the world. Iran is located in the stroke belt and has a high age-adjusted stroke incidence rate. In this multistep prospective qualitative study, we aimed at investigating the status and challenges of stroke management in Iran and explore possible solutions.

**Methods:** In the first and second phase, we attempted to define the status of stroke management in Iran by searching the relevant literature and conducting semi-structured interviews with health-care providers in thirteen hospitals located in seven large cities in Iran. In the third phase, we tried to recommend possible solutions based on international standards and experience, as well as interviews with stroke experts in Iran and the United States.

**Results:** Little public awareness of stroke symptoms and its urgency, low prioritization for stroke management, and an inadequate number of stroke-ready hospitals are some of the major obstacles toward timely treatment of stroke in Iran. Every hospital in our pool except two hospitals had guideline-based algorithms for the administration of intravenous thrombolysis. However, there was no single call activation system for stroke alert. Data from some of the centers showed that hospital arrival of stroke patients to final decision-making took 116-160 minutes. Although there were four endovascular programs in our target areas, there was no center with 24-hour coverage.

**Conclusion:** There are many challenges as well as potentials for improvement of stroke care in Iran. Improving public knowledge of stroke and establishing an organized and comprehensive stroke program in the hospitals will improve acute stroke management in Iran. The Iranian ministry of health should define and advocate the establishment of stroke centers, track the rate of death and disability from stroke, introduce pathways to improve the quality of stroke care through national data monitoring systems, and eliminate disparities in stroke care.

## Introduction

Stroke is among the leading causes of death and permanent disability worldwide.^   1 ^ There is no national stroke registry in Iran. Therefore, a disparity exists between the reported regional incidences of stroke, ranging from 22 to 140 stroke patients per 100000 populations. ^2^^,^^3^ Compared with some of the developed countries, ischemic stroke occurs approximately one decade earlier among Iranian people and leads to a higher rate of mortality.^2^^,^^4^ 

Timely thrombolytic therapy with intravenous tissue plasminogen activator (IV-tPA) is an effective treatment for acute ischemic stroke. ^5^^,^^6^ Patients who are treated in a stroke center have a higher survival rate and better functional outcome.^7^^–^^9^ The Brain Attack Coalition in the United States has accordingly proposed primary and comprehensive stroke centers, in addition to acute stroke-ready hospitals to increase the quality of care for stroke patients. ^10^^–^^13^ 

***Iranian healthcare system:*** Iran provides health-care services to its 79 million citizens through public and private sectors. The Iranian constitution entitles Iranians to basic healthcare, and the Ministry of Health and Medical Education has specific mandate to provide and monitor healthcare delivery.^   14 ^ Public medical schools as delegates of the Ministry of Health are responsible for providing healthcare services and medical education in each province. There is also a national healthcare network that provides basic healthcare services in rural and remote areas in Iran.^   14 ^ In Iran, public hospitals are usually affiliated with medical schools and are the primary provider of specialty and higher levels of care. These hospitals are generally the host of pilot studies for national decision making by Iranian Ministry of Health. Nongovernmental charitable organizations also operate a tiny number of specialty hospitals mainly in the major metropolitan areas in Iran. There is a considerable disparity in accessibility of advanced healthcare services between urban and rural areas in Iran.^   15 ^

***Stroke care in Iran:*** Although there has been a significant improvement in stroke care in Iran, reports indicate that the utilization of intravenous tissue plasminogen activator (IV-tPA) in Iran is lower than many other countries.^   16 ^ At the same time, there is no fine-tuned national standard for developing stroke centers in Iran. The Iranian Ministry of Health has recently declared that the prevention and treatment of stroke is a national health priority.^   17 ^

The aim of this prospective qualitative study was to investigate the status and challenges of stroke management in Iran. We recommended adaptive solutions to improve the quality of stroke care in Iran.

## Materials and Methods

We conducted this three-phase study from June to December 2015.

Phase one: In the first step, we retrieved published studies on stroke in Iran through PubMed and Iranmedex (Table 1) with no date/time, language, document type, and publication status limitations on July 31, 2015. We selected the keywords through controlled vocabulary Medical Subject Headings (MeSH), literature review and experts’ opinion. We also examined reference sources of relevant studies. In addition, we browsed portals of Iranian Medical Council, Iranian Ministry of Health, medical universities and news agencies with open source electronic archives for stroke-related news, statistics, and strategies.

We included all the studies containing any data on stroke in Iran. The primary focus was centered around providing stroke care, stroke team, public awareness and education, therapies and management, rehabilitation, prevention, protocols, monitoring, epidemiology, burden, morbidity, and mortality. Studies focusing on the pathophysiological aspects of stroke such as reports of cellular, molecular and immunologic events, genetic variations, neuroimaging findings and classifications, animal studies and studies lacking any information regarding Iran were excluded from this review.

Phase two: For the second phase of this study, we conducted semi-structured, targeted, one-to-one interviews with different practitioners and care providers in thirteen tertiary public hospitals in seven large Iranian cities. Our survey included a series of questions related to the requirements of acute stroke management (Table 2). To design the questionnaire, we reviewed the United States practice standards^18^^–^^22^ and the concept of stroke unit in Europe.^7^^,^^23^^,^^24^ We also reviewed the details of two city-wide stroke protocols in Memphis, Tennessee, and Birmingham, Alabama, United States.

**Table 1:** Search strategies

A. http://www.ncbi.nlm.nih.gov/
1- ("stroke"[MeSH Terms] OR "stroke"[All Fields]) OR ("acute"[All Fields] AND "stroke"[All Fields]) OR ("acute stroke"[All Fields]) AND ("organization and administration"[MeSH Terms]) OR ("organization"[All Fields] AND "administration"[All Fields]) OR "organization and administration"[All Fields] OR "management"[All Fields] OR "disease management"[MeSH Terms] OR ("disease"[All Fields] AND "management"[All Fields]) OR "disease management"[All Fields] OR "Brain Ischemia"[MeSH] OR "Cerebral Infarction"[MeSH] OR "Hypoxia-Ischemia, Brain"[MeSH] OR "Ischemic Attack, Transient"[MeSH] OR "Infarction, Posterior Cerebral Artery"[MeSH] OR "Brain Stem Infarctions"[MeSH] OR "Infarction, Middle Cerebral Artery"[MeSH] OR ("Infarction, Anterior Cerebral Artery"[MeSH])
2- ("stroke"[MeSH Terms]) OR ("stroke"[All Fields] AND unit[All Fields])
3- ("stroke"[MeSH Terms]) OR ("stroke"[All Fields] AND team[All Fields])
4- (public[All Fields]) AND ("awareness"[MeSH Terms] OR "awareness"[All Fields])
5- (public[All Fields]) AND ("education"[Subheading] OR "education"[All Fields] OR "educational status"[MeSH Terms]) OR ("educational"[All Fields] AND "status"[All Fields]) OR ("educational status"[All Fields] OR "education"[All Fields] OR "education"[MeSH Terms])
6- ("therapy"[Subheading] OR "therapy"[All Fields] OR "therapeutics"[MeSH Terms] OR "therapeutics"[All Fields]) AND ("stroke"[MeSH Terms] OR "stroke"[All Fields])
7- ("tissue plasminogen activator"[MeSH Terms] OR tissue plasminogen activator[Text Word] OR thrombolysis[All Fields]) OR ( "Thrombectomy/adverse effects"[MeSH] OR "Thrombectomy/contraindications"[MeSH] OR "Thrombectomy/education"[MeSH] OR "Thrombectomy/epidemiology"[MeSH] OR "Thrombectomy/instrumentation"[MeSH] OR "Thrombectomy/legislation and jurisprudence"[MeSH] OR "Thrombectomy/methods"[MeSH] OR "Thrombectomy/mortality"[MeSH] OR "Thrombectomy/nursing"[MeSH] OR "Thrombectomy/organization and administration"[MeSH] OR "Thrombectomy/pharmacology"[MeSH] OR "Thrombectomy/psychology"[MeSH] OR "Thrombectomy/standards"[MeSH] OR "Thrombectomy/statistics and numerical data"[MeSH] OR "Thrombectomy/therapeutic use"[MeSH] OR "Thrombectomy/therapy"[MeSH] OR "Thrombectomy/trends"[MeSH] OR "Thrombectomy/utilization"[MeSH] )
8- ("stroke"[MeSH Terms] OR "stroke"[All Fields]) AND ("rehabilitation"[Subheading] OR "rehabilitation"[All Fields] OR "rehabilitation"[MeSH Terms])
9- ("stroke"[MeSH Terms] OR "stroke"[All Fields]) AND ("prevention and control"[Subheading]) OR ("prevention"[All Fields] AND "control"[All Fields]) OR ("prevention and control"[All Fields] OR "prevention"[All Fields])
10- (public[All Fields]) AND ("education"[Subheading] OR "education"[All Fields] OR "educational status"[MeSH Terms]) OR ("educational"[All Fields] AND "status"[All Fields]) OR ("educational status"[All Fields] OR "education"[All Fields] OR "education"[MeSH Terms])
11- ("stroke"[MeSH Terms] OR "stroke"[All Fields]) AND ("nurses"[MeSH Terms] OR "nurses"[All Fields] OR "nurse"[All Fields])
12- ("stroke"[MeSH Terms] OR "stroke"[All Fields]) AND (protocol[All Fields])
13- ("stroke"[MeSH Terms] OR "stroke"[All Fields]) AND (monitoring[All Fields])
14- ("stroke"[MeSH Terms] OR "stroke"[All Fields]) AND ("epidemiology"[Subheading] OR "epidemiology"[All Fields] OR "epidemiology"[MeSH Terms])
15- ("stroke"[MeSH Terms] OR "stroke"[All Fields]) AND ("epidemiology"[Subheading] OR "epidemiology"[All Fields] OR "incidence"[All Fields] OR "incidence"[MeSH Terms])
16- ("stroke"[MeSH Terms]) OR ("stroke"[All Fields] AND burden[All Fields])
17- ("stroke"[MeSH Terms] OR ("stroke"[All Fields]) AND ("mortality"[Subheading] OR "mortality"[All Fields] OR "mortality"[MeSH Terms])
18- ("stroke"[MeSH Terms] OR "stroke"[All Fields]) AND ("epidemiology"[Subheading] OR "epidemiology"[All Fields] OR "morbidity"[All Fields] OR "morbidity"[MeSH Terms])
19- ("iran"[MeSH Terms] OR "iran"[All Fields]) OR (Iranian[All Fields]OR Persian[All Fields]) OR (Tehran[All Fields] OR Shiraz[All Fields] OR Mashhad[All Fields] OR Tabriz[All Fields] OR Isfahan[All Fields] OR Ahwaz[All Fields] OR Zahedan[All Fields]) OR ("middle east"[MeSH Terms] OR "middle"[All Fields] AND "east"[All Fields] OR "middle east"[All Fields])
20- (#1) AND (#2 OR #3 OR 3# OR #5OR #6 OR #7 OR #8 OR #9 OR #10 OR #11 OR #12 OR #13 OR #14 OR #15 OR #16 OR #17 OR #18) AND (#19)
B. http://iranmedex.ir/
1- stroke OR infarction OR cerebrovascular event OR CVA OR brain ischemia
2- stroke unit OR stroke team OR stroke awareness OR therapy OR thrombolysis OR thrombectomy OR rtPA OR tissue plasminogen activator OR rehabilitation OR prevention OR public education OR nurse OR protocol OR monitoring OR epidemiology OR mortality OR morbidity
3- #1 AND #2
4- Corresponding terms in Persian

**Table 2:** The questions for semi-structured interviews and collective results: evaluating the current state of acute stroke management

**Requirements of acute stroke management**	**Hospital met the ** **requirement (%)**
EMS stroke screening protocol, early management, and sending pre-notification to receiving hospital	0
Fast and efficient triage system in the ED for patients with stroke-like symptoms	0
Single call activation system for stroke alerts	0
On-call neurologist available for urgent consultation	100 (Neurology house staff)
Active 24-hour stroke program (trained nurses, available pharmacist) to deliver emergency intravenous therapies to eligible stroke patients	0
24-hour access to CT scans for an urgent scan within 30 minutes of a patient’s arrival to emergency room	100 (No stroke protocol exist)
24-hour access to urgent and basic laboratory studies in emergency room	100 (No stroke protocol exist-results delayed in all centers)
Active 24-hour program to deliver emergency endovascular treatment to eligible stroke patients or a referral system in place to transfer the patient to an appropriate center	31 (Not available 24-h)
Guideline-based algorithms, order sets, tPA dosing charts in emergency rooms	85
Periodic educational programs in stroke care for ED staff	38
Inpatient facility to admit patients with stroke. In the absence of intensive care unit, a referral system should be available to transfer the patients to appropriate units	100
Patient care and daily visit by a neurologist and a multidisciplinary team	100 (By neurology team-no multidisciplinary team available)
Access to advance imaging for further stroke diagnosis	85
Cardiologist and cardiac imaging facilities	100
Active 24-hour program to deliver emergency neurosurgical treatment to eligible stroke patients or a system in place to transfer the patient to an appropriate center	100
Trained stroke nursing staff in stroke service	0
Periodic educational programs in stroke care for stroke service nursing staff	8
Established and organized palliative care/end of life pathway	0
Stroke nurse-coordinator	0
Ongoing commitment reflected in the quality assurance measures for better stroke care	0
Stroke data bank to collect measures applicable to quality assurance and better patient care	0
Stroke Rehabilitation program in the hospital	0
Rehabilitation hospital or outpatient rehabilitation unit with specialized stroke program	0
Special training course for patient, family, and caregiver to focus on secondary prevention and rehabilitation	0
Organized approach to follow-up visit by a neurologist or stroke rehab specialist	100 (The first neurologic visit following discharge)
Organized support group for patient and family	0

EMS: Emergency medical services; CT scan: Computed tomography; tPA: Tissue plasminogen activator; ED: Emergency department; Total number of hospitals evaluated in this study: 13

To choose the hospitals, we stratified the Iranian population into seven strata according to the geographical and population density distribution. Then, we chose the most populated city in each stratum (two-stage clustered sampling method). The selected cities are among the twelve most populated metropolitans in Iran-altogether they include about 50% of the Iranian population.^   25 ^

Only a small number of the major cities in Iran have private hospitals, and the Iranian national referral network does not include these private hospitals. In addition, many healthcare insurance providers do not cover for services offered in private centers. Consequently, negligible percent of stroke patients visit private hospitals. Therefore, we only selected thirteen tertiary public hospitals-seven hospitals in Tehran and one hospital in each of the following cities: Isfahan, Shiraz, Tabriz, Mashhad, Ahwaz and Zahedan (Iran).

We used judgment selection method followed by snowball sampling to select and interview providers and other healthcare personnel in each hospital. 

The interviews were performed either in-person or by phone. Each interview lasted between 30 to 40 minutes. The interviews were done in a neutral and collaborative setting. The identity of interviewees remained confidential.

Phase three: We examined our results and tried to recommend some targeted and adaptive solutions for stroke care in Iran. To achieve that, we consulted with several local experts and policy makers, in addition to a few stroke experts in the United States. 

We analyzed the data using the first coding process through initial coding. This type of coding was chosen to examine, compare, and search for similarities and differences throughout the data. The second level coding was pattern coding to explain major themes underneath the segments of the data and to understand the relationships.

## Results

**Figure 1:**
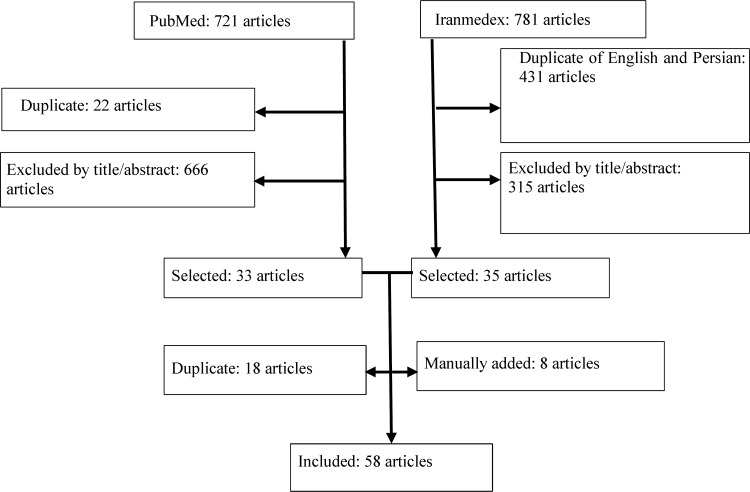
Literature search and selection of studies (phase one)

***Review of literature:*** We reviewed more than 1500 news reports and research articles. Fifty-eight articles were selected and adequately studied (Figure 1). The most prominent obstacle toward timely treatment of stroke was a lack of priority for acute stroke management and low public awareness of stroke symptoms and its urgency. Almost 95% of the stroke patients who had visited a tertiary stroke care in Tehran lacked any awareness and prior education about stroke symptoms.^   26 ^ Studies from Iran showed that more than 17 percent of Bushehr’s residents^   27 ^ and 48.7% of the stroke patients admitted to a referral center in Tehran^   28 ^ could not name even one stroke risk factor. The gap between the knowledge of stroke risk factors and behavior^   4 ^ and wrong attitude toward availability, efficiency, and affordability of the stroke management^4^ were also reported. 

Reports from Tabriz, Mashhad, and Isfahan demonstrated that less than 30% of the stroke patients arrived at the hospital within three hours of symptoms onset. Up to 40% of ischemic stroke patients did not present to the emergency department (ED) on the day of symptom onset.^16^^,^^29^^-^^31^ It was also reported that 45% to 86% of the patients who arrived within 3-hour window of symptom onset missed thrombolytic therapy due to the lengthy diagnostic process.^26^^,^^30^^,^^32^^–^^34^ 

***Semi-structured interviews:*** For the second phase of the study, we interviewed a total of 76 practitioners and care providers (Table 3) in thirteen public tertiary centers in seven large metropolises in Iran. All thirteen hospitals in our cohort had a 24-hour neurology service and a neurology resident available for urgent consults. Every hospital had access to 24-hour computed tomography (CT) scan; however, immediate access to advanced imaging including CT angiogram, perfusion imaging or magnetic resonance imaging (MRI) was limited in all selected hospitals. Every hospital in our pool except two hospitals had guideline-based algorithms for the administration of intravenous thrombolysis. There was no single call activation system for stroke alert. Although there were four endovascular programs in our target areas, there was no center with 24-hour coverage.

**Table 3:** The study participants (second phase) in thirteen hospitals

**Participants**	**n**	**Participants**	**n**
Neurologists	24	Pharmaceutical representatives	2
Neurosurgeons	7	ED physicians	6
EMS staff	11	Hospital receptionists	3
Cardiologists	2	Imaging and laboratory staff	3
Radiologists	3	Administrators	9
Intensivists	3	Triage staff	5
Nurses	16	Case managers	4

ED: Emergency department; EMS: Emergency medical services; Some participants owned more than one responsibility.

There was no stroke registered or trained nurses in the ED, stroke nurse-coordinator, stroke navigator, systematic data monitoring or feedback system in any of our surveyed hospitals. Only one hospital offered annual stroke training courses for nursing staff. Table 2 includes the percentage of hospitals who met each of our defined requirements for acute stroke management. 

Almost every ED in our cohort was suffering from the delayed assessment of stroke patients and prolonged “door to needle” time for IV-tPA. Data from some of the centers showed that hospital arrival of stroke patients to final decision-making took 116-160 minutes. 

There was not a regular and formal training as well as protocol regarding stroke screening and pre-hospital stroke management at least in our surveyed centers. Every hospital suffered from a lack of stroke rehabilitation programs and organized palliative care service.

## Discussion

Our study, similar to the other studies^35^^-^^39^ suggests that delayed hospital presentation in addition to the lack of an organized and comprehensive stroke program in the hospitals are the biggest obstacles to receiving proper acute stroke treatment. 

Several studies published since 2000, reporting a median delay of symptom onset to ED arrival, indicated that the 50^th^ percentile for delay occurred between 3 and 4 hours^   35 ^ with 14%-48% arrival within two hours and 15%-60% arrival of the patients within three hours after symptom onset.^   39^

The rate of early presentation to the ED after stroke is significantly lower in Iran. Although lack of health insurance can be associated with delays in seeking emergency care,^   40 ^ it does not seem to be a significant factor in Iran especially after extending universal health insurance (so-called “RouhaniCare”) to all Iranians. Limited public knowledge of stroke warning symptoms^   26 ^ and false attitude toward availability and affordability of the stroke management^   4 ^ had been reported as possible causes. In addition, similar to other health services, a disparity exists in stroke care in Iran, and except large and industrialized cities, other small towns do not have adequate infrastructures or trained personnel for management of patients with stroke.^   15 ^

Emergency medical services (EMS): The pre-hospital workplace 

EMS are the integral component of stroke centers by their vital role in rapid transport of the stroke patients to designated facilities.^   12 ^ Based on stroke statements and guidelines, the average time between notification and ambulance arrival should be less than eight minutes, with the application of alternatives such as air ambulance when it takes more than one hour to access the medical center.^   41 ^ In Iran, rapid transport of the patients is challenging.^42^^,^^43^ Moreover, our study showed that EMS staff in our studied pool did not receive enough training about stroke, and they might be confused about their role in stroke assessment and care. 

In-hospital stroke management: from acute treatment to rehabilitation

Improper triage is another time sparing factor in stroke management.^44^^-^^46^ Although all the centers had a 24-hour neurology service, several physicians in our study reported that a considerable number of patients were improperly triaged in the ED. 

Other reasons for delay were late imaging or laboratory evaluations, prioritizing acute stroke patients or overwhelmed EDs with critically ill patients and high demands for diagnostic modalities. The ED was also suffering from a lack of resources. Despite these limitations, it is expected that adaptation of stroke alert and sending pre-notification to the destination hospital accelerate the treatment. 

Performance monitoring and feedback system: an essential step for quality improvement

Data collection and care performance surveillance system for the stroke care pathway is essential. Introducing pathways to improve the quality of stroke care through national data monitoring systems is imperative. Unfortunately, there is no national data surveillance system in Iran. There was also no comprehensive data monitoring or feedback system in our participating centers.

What are the solutions?

The most important step to improve the management of stroke in Iran could be to put stroke as a top healthcare priority. Studies in other countries^47^^,^^48^ and also experience in the management of myocardial infarction in Iran^49^^,^^50^ showed that by making stroke care a top priority and using mass media to increase awareness, many of the cited impediments could be solved. During the last phase of our study, we tried to recommend some targeted and adaptive solutions for stroke care in Iran based on several local and international experts’ opinions. Table 4 summarizes some of the recommendations to improve stroke management in Iran. 

**Table 4:** The obstacles and possible solutions for stroke care in Iran

**Obstacles**	**Possible solution**
Public awareness Lack of public awareness about stroke symptoms and its urgency	Campaigns to increase community awareness Public education in health houses and health centers: face-to-face education with considering language and cultural considerations Mass media: television, radio, outdoor banners, newsletters, the Internet Group educations for relatives of stroke victims in the hospital Special programs for the elderly population in parks, senior recreation centers, religious centers, etc. Education for school children and their family, Banners, and flyers in health centers, physician office, etc.
Pre-hospital assessment and care Traffic congestion and delayed EMS arrival Lack of or inefficient screening protocol and early stroke assessment and management by EMS personnel Lack of public awareness to yield the right of way to an emergency vehicle	Increase EMS resources Provide regular education for emergency call attendances to identify possible cases of stroke through the phone conversation and rapidly dispatch the EMS team Prioritize stroke response in EMS system Improve public awareness to yield the right of way when approached by an emergency vehicle More training for EMS staff about early stroke recognition, recognizing possible cases of large vessel occlusion, and their role in early stroke assessment and care Evaluate the feasibility and effectiveness of Stroke Emergency Mobile in largely populated cities like Tehran. Stroke mobile includes a CT scanner and point-of-care laboratory installed in a fully equipped ambulance
In-hospital acute stroke management Incorrect or delayed assessment of stroke patients in triage Long “Door To Needle” time secondary to lack of fast and efficient triage system, lack of single activation call system, delayed imaging Lack of emergency guideline-based algorithms, trained stroke nurses, urgent access to advanced imaging, 24-hour endovascular program Lack of regular educational program for ED personnel Lack of organized data and performance monitoring and feedback system for quality improvement	Install several easy-to-read wall posters in the ED waiting room to draw patients’ attention to the signs of stroke requiring them to alert the triage nurse immediately Establish standard operating procedures and protocols to triage stroke patients rapidly Provide general education for triage nurses Enable triage nurses to activate stroke alert Establish a team-based approach in the ED and train professional stroke registered nurses Provide organized and professional stroke team at the hospital with a focused goal Single Call Activation System: a single call should activate the entire stroke team at the hospital Provide rapid triage protocol for inpatient and early stroke team notification at the hospital Every hospital medical staff should be able to activate stroke alert
	Mobilize the imaging and laboratory facilities by the aid of activated stroke alert or pre-notification system Performing CT scan (or MRI) within 25 minutes of arrival and complete interpretation of the CT scan within 45 minutes of arrival Rapidly recognize patients with large vessel occlusion and alert the interventional team Provide regular educational programs for ED staff Have a protocol in place for the rapid transfer of patients to a tertiary care center, if needed Establish an organized data monitoring and feedback system for quality improvement evaluation
Inpatient stroke management Lack of trained stroke nursing staff, routine training for nursing staff, and timely physical, occupational and speech therapist evaluation and a multidisciplinary team round. Access to advanced neurological and cardiovascular imaging/testing can be a challenge Lack of coordinated palliative care/end of life pathway Lack of organized data and performance monitoring and feedback system for quality improvement	Admit stroke patients directly to a stroke unit or stroke service under the care of a stroke specialist and a multidisciplinary team. Access to a neurological ICU Easy access to advance imaging for further investigation of stroke patients Provide routine training for nursing staff to provide high-quality nursing care Daily monitoring and documentation of NIH stroke scale Perform swallowing screening assessment on admission by appropriately trained and competent staff Nutritional screening assessment performed within 24 hours of admission Protocol for the promotion of bladder and bowel continence including a policy to avoid urinary catheters Provide established protocols for the prevention and treatment of common complications Establish an organized palliative care/end of life pathway
Rehabilitation program Lack of a comprehensive in-patient, out-patient stroke program in all studied centers	Establish a designated stroke rehabilitation inpatient unit All medically stable patients with stroke should be transferred from the stroke service without delay Screen for cognitive deficits, visual neglect, attention deficits and emotional problems and have access to a specialist in clinical psychology Involve families and caregivers in day-to-day care and rehabilitation Encourage patient and family in secondary stroke prevention and change of lifestyle (nutrition, weight loss, medicine compliance, physical activity) Establish a protocol for patients’ follow-up visit Organize stroke support groups for patients and families Organize the rehab protocol based on patient, family, and community
Other challenges Disparity in stroke care in Iran Lack of national guideline for primary and comprehensive stroke center designation Lack of a pathway to improve the quality of stroke care through national data monitoring systems	Develop telehealth capabilities for remote stroke diagnosis and treatment Develop programs for underrepresented minority populations and women Public-private partnerships and shared resources Develop and certify primary stroke center policy through the national legislative system: examples include primary stroke center designation through a national program, EMS protocols, or hospital bypass policies Monitor, and improve the quality of and access to care for stroke patients from the onset of stroke symptoms through the rehabilitation Track the rate of death and disability from acute stroke Monitor and eliminate disparities in stroke care Increase the epidemiological knowledge of stroke in Iran Introduce pathways to improve the quality of stroke care through national data monitoring systems

EMS: Emergency medical service; CT scan: Computed tomography scan; ED: Emergency department; MRI: magnetic resonance imaging; ICU: Intensive care unit; NIH: National Institutes of Health

The Iranian primary healthcare system is a unique system that was established to improve access to healthcare for the disadvantaged and reduce the gap between the urban and rural areas. The smallest unit of Iranian healthcare system is called “health house.” Health houses are designed to cover a target population of about 1500 in rural areas with careful attention to their cultural and social characteristics. We believe that health houses can have a crucial role in stroke education and support in remote regions.

It is evident that stroke centers can further improve stroke patients’ outcome. Patients treated in stroke centers are 11% less likely to die, 11% less likely to be in institutional care, and 16% more likely to live at home one year after their stroke than patients treated in other hospitals.^51^ Therefore, defining national criteria for stroke center is an essential step in Iran. We believe that many of the medical centers in Iran have the potential to become a stroke center.

Our study had some limitations. In this study, we only included thirteen tertiary centers in seven large cities. Some other hospitals in smaller cities might have more limitations in terms of infrastructure and personnel required for proper stroke management. We performed some of our interviews by phone. It might be more difficult to connect and build rapport in a meaningful way over the phone. Although on many occasions we interviewed more than one individual from the same department, we did not have any other ways to confirm the accuracy of individual responses.

## Conclusion

There are many challenges as well as potentials for improvement of stroke care in Iran. Improving public knowledge of stroke and establishing an organized and comprehensive stroke program in the hospitals will improve acute stroke management in Iran. The Iranian ministry of health should define and advocate the establishment of stroke centers, track the rate of death and disability from stroke, introduce pathways to improve the quality of stroke care through national data monitoring systems and eliminate disparities in stroke care.

## Notes:

**How to cite this article:** Shahjouei S, Bavarsad-Shahripour R, Assarzadegan F, Rikhtegar R, Mehrpour M, Zamani B, et al. Acute management of stroke in Iran: Obstacles and solutions. Iran J Neurol 2017; 16(2): 62-71.
